# CXCL2/CXCR2 pathway regulates CD169^high^ large macrophages to promote progression of malignant pleural effusion from lung adenocarcinoma

**DOI:** 10.3389/fmed.2026.1943314

**Published:** 2026-09-09

**Authors:** Sitong Chen, Chunyan Li, Yuezhang Sun, Yanyan Huang, Guifu Gong, Yuxuan Bao, Liyun Xu

**Affiliations:** 1 Department of Clinical Laboratory, Zhoushan Hospital, Wenzhou Medical University, Zhoushan, Zhejiang, China; 2 Cellular and Molecular Biology Laboratory, Zhoushan Hospital, Wenzhou Medical University, Zhoushan, Zhejiang, China; 3 Department of Cardiothoracic Surgery, Zhoushan Hospital, Zhejiang University School of Medicine, Zhoushan, Zhejiang, China; 4 First Affiliated Hospital of Wenzhou Medical University, Wenzhou, China; 5 Department of Pulmonary and Critical Care Medicine, Wenzhou Medical University, Zhoushan, Zhejiang, China

**Keywords:** CD169^+^ macrophages, CXCL2, CXCR2, lung adenocarcinoma, malignant pleural effusion (MPE)

## Abstract

**Background:**

Malignant pleural effusion (MPE) correlates with poor prognosis. Tumor-associated macrophages dominate the immune microenvironment and govern MPE progression, while the functional heterogeneity of macrophage subsets in MPE remains poorly understood. CD169^+^ macrophages exhibit context-dependent tumor-regulatory functions in various malignancies, yet their phenotypic characteristics and biological roles in MPE remain uncharacterized.

**Methods:**

CD169⁺ macrophages and their CD169^low^ small and CD169^high^ large subsets were characterized in PPE, IPE, and lung adenocarcinoma-associated MPE, as well as in a murine MPE model, by flow cytometry. Their associations with MPE control were analyzed. The effects of CXCL2/CXCR2 signaling on macrophage migration, proliferation, and apoptosis were evaluated using recombinant human CXCL2 and the CXCR2 inhibitor SB225002 *in vitro*, and the therapeutic effects of CXCR2 inhibition were assessed *in vivo*.

**Results:**

CD169⁺ macrophages, including CD169^low^ small and CD169^high^ large macrophages, were significantly enriched in lung adenocarcinoma-associated MPE (*P* < 0.05). Only CD169^high^ large macrophages were negatively associated with MPE control (*P* < 0.05) and exhibited an immunosuppressive phenotype. Macrophages were the predominant CXCL2-expressing population in single-cell analysis. CXCL2 promoted macrophage migration, whereas CXCR2 inhibition with SB225002 attenuated this effect without affecting macrophage proliferation or apoptosis. *In vivo*, SB225002 reduced pleural effusion volume, CD169^high^ macrophage accumulation, and tumor burden.

**Conclusions:**

We identified a distinct population of CD169^high^ large macrophages that is enriched in lung adenocarcinoma-associated MPE and exhibits an immunosuppressive phenotype. CXCL2/CXCR2 axis promotes macrophage migration and contributes to the accumulation of CD169^high^ macrophages in the MPE microenvironment, thereby facilitating MPE progression. These findings provide new insights into the myeloid-cell regulatory mechanisms underlying MPE and may represent a potential therapeutic strategy for lung cancer-associated MPE.

## 1 Introduction

Malignant pleural effusion (MPE) is a frequent and debilitating complication of advanced malignancies, most commonly lung adenocarcinoma, and is associated with dismal prognosis and limited therapeutic options (1). The pathogenesis of MPE involves complex crosstalk among tumor cells, the pleural vasculature, and immune cells, culminating in increased vascular permeability, neoangiogenesis, and intrapleural inflammation (1). Tumor-associated macrophages (TAMs) constitute the dominant immune cell population in MPE, and accumulating evidence indicates that their abundance and polarization state are critical determinants of disease progression and patient survival (1, 2). In particular, CD163^+^ M2-polarized macrophages have been consistently associated with an immunosuppressive and pro-angiogenic phenotype in MPE, and elevated counts of these cells in pleural fluid predict impaired survival (2). Nevertheless, despite the well-recognized dominance of TAMs in MPE, the full spectrum of macrophage heterogeneity within this unique tumor microenvironment (TME) remains incompletely characterized. Most prior investigations have relied on the binary M1/M2 classification framework or focused predominantly on CD163^+^ M2 cells, thereby neglecting the existence and potential contributions of other functionally distinct macrophage subsets. This knowledge gap is particularly salient given that recent single-cell transcriptomic studies have uncovered a remarkable diversity of macrophage populations in MPE, including C1q^+^ and FOLR2^+^ subsets, each with unique transcriptional programs and immunomodulatory functions (3–5). These findings underscore the need to move beyond the M1/M2 paradigm and systematically characterize previously underappreciated macrophage subsets in MPE.

Among the emerging macrophage subsets of interest, CD169^+^ (Siglec-1^+^) macrophages have attracted increasing attention in tumor immunology. CD169 is a sialic acid-binding immunoglobulin-like lectin that is constitutively expressed by tissue-resident macrophages in the marginal zone of the spleen and the subcapsular sinus of lymph nodes, with lower-level expression detected in macrophages of the lung, liver, and intestine (6, 7). In the context of cancer, CD169^+^ macrophages exhibit context-dependent and sometimes opposing functions. In tumor-draining lymph nodes, CD169^+^ macrophages play a predominantly antitumorigenic role by capturing tumor-derived extracellular vesicles, cross-presenting tumor antigens to CD8^+^ cytotoxic T cells, and restraining the generation of tumor-promoting B cell responses (6, 8). Conversely, in certain primary tumor tissues, CD169^+^ macrophage infiltration has been associated with worse prognosis, suggesting that the TME can reprogram these cells toward protumorigenic phenotypes (6). A recent study in glioblastoma demonstrated that blood monocyte-derived CD169^+^ macrophages can exert potent antitumor activity by producing pro-inflammatory chemokines that recruit T cells and NK cells, and by facilitating phagocytosis of apoptotic tumor cells (8). These findings collectively indicate that the functional orientation of CD169^+^ macrophages is exquisitely sensitive to tissue-specific microenvironmental cues. Despite this growing body of evidence, the presence, phenotypic characteristics, and functional role of CD169^high^ macrophages in MPE have never been systematically investigated. It remains entirely unknown whether CD169^high^ macrophages accumulate in the pleural cavity of lung adenocarcinoma patients, and if so, whether they adopt an antitumorigenic or protumorigenic phenotype in this immunosuppressive, hypoxic, and inflammation-rich microenvironment. Addressing this gap is essential for understanding the full immunological landscape of MPE and for identifying novel macrophage-centered therapeutic targets.

The recruitment and polarization of macrophages in the TME are governed by a complex network of chemokine–chemokine receptor axes, among which the CXCL2/CXCR2 pathway has emerged as a critical regulator (9). CXCL2 is an ELR^+^ CXC chemokine that signals predominantly through the CXCR2 receptor and is well established for its roles in neutrophil chemotaxis, myeloid-derived suppressor cell (MDSC) recruitment, and macrophage polarization (9). In the context of lung adenocarcinoma, elevated CXCL2 expression and CXCR2 activation have been associated with advanced disease, poor differentiation, and resistance to therapy, and pharmacological CXCR2 inhibition has been shown to suppress tumor progression and enhance chemosensitivity in preclinical models (9, 10).

Building on these observations, we propose that the CXCL2/CXCR2–CD169 macrophage axis represents a previously unrecognized pathogenic pathway in lung adenocarcinoma-associated MPE. In this study, we quantified the proportions and phenotypic marker expression of CD169^+^ macrophages in clinical specimens from postoperative pleural effusion (PPE), inflammatory pleural effusion (IPE), and MPE patients, as well as in a murine MPE model. We further analyzed the correlation between CD169^+^ macrophage profiles and the clinical control rate of pleural effusion. Additionally, we investigated the expression patterns of chemokine receptors and their ligands on CD169^+^ macrophages in MPE, aiming to clarify the mechanism by which CD169^high^ large macrophages drive MPE development. This study seeks to provide novel theoretical insights into the immune regulatory mechanisms of MPE and offer innovative targets for the development of macrophage-based precise therapeutic strategies for MPE.

## 2 Materials and methods

### 2.1 Human pleural effusion samples

From January 2023 to October 2024, 10 fresh PPE samples from patients with stage IA lung adenocarcinoma, 10 fresh IPE samples, and 32 fresh MPE samples from lung adenocarcinoma were collected at Zhoushan Hospital (Zhoushan, Zhejiang, China).

The inclusion criteria for PPE: (1) No pleural effusion occurred before surgery. (2) No treatment was received before surgery. (3) Pleural effusion occurred after surgery. (4) Pathological examination after surgical resection confirmed stage IA lung adenocarcinoma. The exclusion criteria for PPE: (1) Patients with severe chronic diseases, tumors, or autoimmune diseases. (2) Patients who received radiotherapy, chemotherapy, or other treatments before surgery. (3) Patients with signs of infection.

The inclusion criteria for IPE: (1) No cancer cells were found in the cytological examination of pleural effusion. (2) Blood tests indicated infection in patients. (3) Clinical diagnosis was pneumonia. The exclusion criteria for IPE: Patients with severe chronic diseases, tumors, or autoimmune diseases.

The inclusion criteria for MPE: (1) Cancer cells were found in the cytological examination of pleural effusion. (2) Primary lung cancer was confirmed by pathological examination after surgical resection, lung puncture, or bronchoscopy biopsy. (3) None of the patients had received radiotherapy, chemotherapy, immunotherapy, etc. before the first diagnosis. The exclusion criteria for MPE: (1) Primary lung cancer complicated with other malignancies, infections, or immune diseases. (2) Secondary lung cancer. (3) Pathological type was *in-situ* adenocarcinoma.

This study was approved by the Ethics Committee of Zhoushan Hospital (No. 2023-093) and informed consent was obtained from all patients.

### 2.2 Animals

Male C57BL6/J mice, aged 6–8 weeks, were obtained from the Beijing Vital River company (Beijing, China). All experimental animals were housed in a pathogen-free animal room with suitable room temperature and humidity. All animal experiments were carried out in accordance with the guidelines for animal experiments. All animal experiments were approved by the Experimental Animal Welfare and Ethics Committee of Zhejiang Free Trade Zone Ruisai Biomedical Technology Co., Ltd.

### 2.3 Isolation of pleural effusion mononuclear cells

Transfer the pleural effusion into a 50 mL centrifuge tube. Then, centrifuge it at 1,000 rpm for 5 min. After that, discard the supernatant. Equal amounts of PBS solution were transferred into 50 mL centrifuge tubes for dilution. An appropriate volume of Ficoll solution (17144003 Cytiva) was added to a new 15 mL centrifuge tube, and then, diluted pleural effusion, which was 2 times the volume of Ficoll solution, was gently added to the upper layer. After centrifugation at 1,000 g for 20 min, the pleural effusion mononuclear cells were located in the white membrane layer. Then, the white membrane layer was transferred to a new 15 mL centrifuge tube, and 5 mL of PBS solution was added. The supernatant was discarded after centrifugation at 1,000 rpm for 5 min, and 5 mL of red blood cell lysis solution (R1010 Solarbio) was added and incubated for 5 min. Finally, the cells were washed with PBS.

### 2.4 Sorting and culturing macrophages

Five milliliters of running buffer (1x PBS solution containing 0.5% BSA and 0.5 mmol/L EDTA) was added to the obtained pleural effusion mononuclear cells and the supernatant was discarded after centrifugation at 1,000 rpm for 5 min. Then, 40 µL of running buffer and 10 µL of biotin antibody (#AB-10373107,Thermo, USA) were added, and the mixture was precooled for 5 min. Next, 30 µL of running buffer and 20 µL of monocyte isolation magnetic beads (#11149D, Thermo, USA) were added, then pre-chill the mixture for another 10 min. Finally, 380 µL of running buffer was added, and the sample was analyzed with an AutoMACS Pro sorting machine (Miltenyi, Germany). Macrophages were resuspended in complete RPMI 1,640 medium containing 10% heat-inactivated fetal bovine serum, 100 U/mL penicillin and 100 *μ*g/mL streptomycin. Cells were plated and incubated at 37 °C in a humidified 5% CO_2_ incubator. Non-adherent cells were discarded after 2 h incubation by gentle PBS washing, leaving adherent cells. The cells were continuously cultured for 5–7 days, and the culture medium was replaced every 2–3 days for subsequent *in vitro* experiments.

### 2.5 Calculation of the effective control rate of MPE

Complete response (CR): The pleural effusion disappeared and was maintained for at least 4 weeks. Partial response (PR): The volume of pleural effusion decreased by 50% and was maintained for at least 4 weeks. Unchanged (NC): The above mentioned criteria for CR or PR were not met. Or, even if there was some improvement, the patient still needed another thoracentesis after 4 weeks of treatment. Progressive disease (PD): The volume of pleural effusion increased. The effective control rate was calculated as the number of patients with CR plus PR divided by the total number of patients.

### 2.6 Establishment of the mouse model of MPE

Twenty male C57BL/6J mice, aged 6–8 weeks and weighing 18–22 g, of SPF grade were selected. Well growing Lewis lung carcinoma cells (purchased from the American Type Culture Collection) in the logarithmic growth phase were collected, and PBS buffer was added to adjust the final cell concentration to 2.5 × 10^6^ cells/mL to obtain a cell suspension. Before inoculation, the skin on the right side of the mouse chest was disinfected with 75% ethanol. A 1 mL syringe was used to draw the cell suspension, which was gently shaken to mix well. The needle was inserted into the pleural cavity from the right chest of the mouse near the mid-axillary line, approximately at the level of the 6th intercostal space. The needle was pointed upward at an angle of 30 degrees to the chest wall, and 0.2 mL of the cell suspension was injected into each mouse.

Model validation method: *in vivo* bioluminescence imaging and photographing of mice were performed on day 7. Prior to the experiment, luciferin substrate was thawed at room temperature. After weighing the mice, the required volume of substrate was calculated at a dosage of 10 mL substrate per 1 g body weight. The corresponding volume of luciferin substrate was then intraperitoneally injected into each mouse. Thirty-five minutes later, the mice were placed into an *in vivo* small animal imaging system to detect the fluorescence signals of pleural effusion and thoracic wall.

### 2.7 *In vivo* experiments

Mice were randomly assigned into experimental groups. The mice in experimental group (*n* = 6) were given a solution containing SB225002 (#HY-16711,MCE, USA), DMSO (SIGMA, Germany), PEG300 (#HY-Y0873, MCE, USA), Tween80 (#HY-Y1891, MCE, USA) and normal saline. SB225002 was in powder form, and the administration concentration was 10 mg/kg. The mice in control group (*n* = 6) were given a solution of DMSO, PEG300, Tween80 and normal saline. The solution was composed of 10% DMSO, 40% PEG300, 5% Tween80, and 45% normal saline. The drugs were administered via intraperitoneal injection. The injection volume was 0.01*m (m represents the animal's body weight in grams) mL per mice. The injections were carried out on the 7th, 10th, and 12th days after the establishment of the mouse model.

### 2.8 Flow cytometry

Before the staining procedure, the Fc receptors of the cells were blocked with TruStain FcX (#101320, BioLegend, USA) for 15 min. The cells were further stained with the antibodies. The staining cells were analyzed using a BD FACSCalibur cytometer (BD Biosciences, San Jose, USA) and the data were analyzed using FlowJo software. The antibodies used are as follows.

7-AAD (#559925, BD Pharmingen, USA); APC anti-human CD45 (#304012, Biolegend, USA); APC anti-human CD14 (#325608, Biolegend, USA); APC anti-human TIM-3 (#345012, Biolegend, USA); APC anti-human TIM-4 (#354008, Biolegend, USA); APC anti-human CD169 (#346008, Biolegend, USA); FITC Mouse Anti-Human CD14 (#555397, BD Pharmingen, USA); FITC Mouse Anti-Human HLA-DR (#555560, BD Pharmingen, USA); FITC anti-human CD45 (#304006, Biolegend, USA); FITC anti-human CD206 (#321104, Biolegend, USA); FITC anti-human CD163 (#333618, Biolegend, USA); PE anti-human TIM-3 (#563422, BD Pharmingen, USA); PE anti-human TIM-4 (#FAB2929P, RD, USA); PE anti-human CD14 (#301805, Biolegend, USA); PE anti-human CD45 (#304008, Biolegend, USA); PE anti-human CD204 (#371904, Biolegend, USA); PE anti-human MS4A4A (#372503, Biolegend, USA); PE anti-human CD169 (#346004, Biolegend, USA); PerCP anti-human CD206 (#321122, Biolegend, USA); PerCP anti-human CD14 (#301847, Biolegend, USA); PerCP anti-human TIM-3 (#345016, Biolegend, USA), APC anti-mouse F4/80 (#AF19999, Elabscience, China)：APC anti-mouse F4/80 (#123116, Biolegend, USA); FITC anti-mouse Ly-6C (#128005, Biolegend, USA); FITC anti-mouse CD169 (#142405, Biolegend, USA); FITC anti-mouse CD169 (#142406, Biolegend, USA); FITC anti-mouse F4/80 (#123107, Biolegend, USA); PerCP anti-mouse CD11b (#AF17268, Elabscience, China); PerCP anti-mouse CD11b (#101229, Biolegend, USA) and their isotypes.

### 2.9 ELISA

CCL1, CCL2, MIF, CXCL2, CXCL3, CXCL5, CXCL8 production in cultured cell supernatant was determined by ELISA kits (eBioscience, China) following the manufacturer's instructions. Absorbance at 450 nm was measured. CCL1, CCL2, MIF, CXCL2, CXCL3, CXCL5, CXCL8 concentrations were calculated from the standard curve.

### 2.10 Single-cell RNA sequencing data analysis

Public single-cell RNA-seq dataset GSE131907 was downloaded from the Gene Expression Omnibus (GEO) database. Raw gene expression matrices were processed and normalized using the Seurat package (v4.2.1) in R software (v4.1.3).

### 2.11 Transwell assay

PBMCs were seeded into the upper chambers of Transwell inserts with 8-μm pore membranes in serum-free medium. 50ug/mL Recombinant human CXCL2 (rhCXCL2) was added to the lower chambers as a chemoattractant, with or without the 20uM CXCR2 antagonist SB225002 (#HY-16711, MCE, USA). After incubation for 4 h at 37 °C in a humidified 5% CO_2_ atmosphere, non-migrated cells were removed from the upper surface of the membrane. Cells that migrated to the lower surface were fixed, stained with crystal violet, and quantified by counting cells in randomly selected microscopic fields. Migration was expressed as the mean number of migrated cells per field.

### 2.12 Statistical analysis

All data were presented as mean ± SD, and *in vitro* experiments were independently repeated at least three times. Statistical analysis was performed using GraphPad Prism 10.0 statistical software. Normality was assessed using the Shapiro–Wilk test. Normally distributed data between two groups were analyzed using Student's *t*-test, while non-normally distributed two-group data were compared via the Mann–Whitney *U*-test. For comparisons involving three or more groups, one-way analysis of variance (ANOVA) was adopted for normally distributed data, and the Kruskal–Wallis *H*-test was utilized for non-normal datasets. Linear regression analysis was performed for correlation analyses of normally distributed variables, whereas Spearman's rank correlation was employed for non-normally distributed data. A *P* value < 0.05 was defined as statistically significant.

## 3 Result

### 3.1 Increased infiltration of CD169^+^ macrophages in MPE of human lung adenocarcinoma

**Figure 1:**
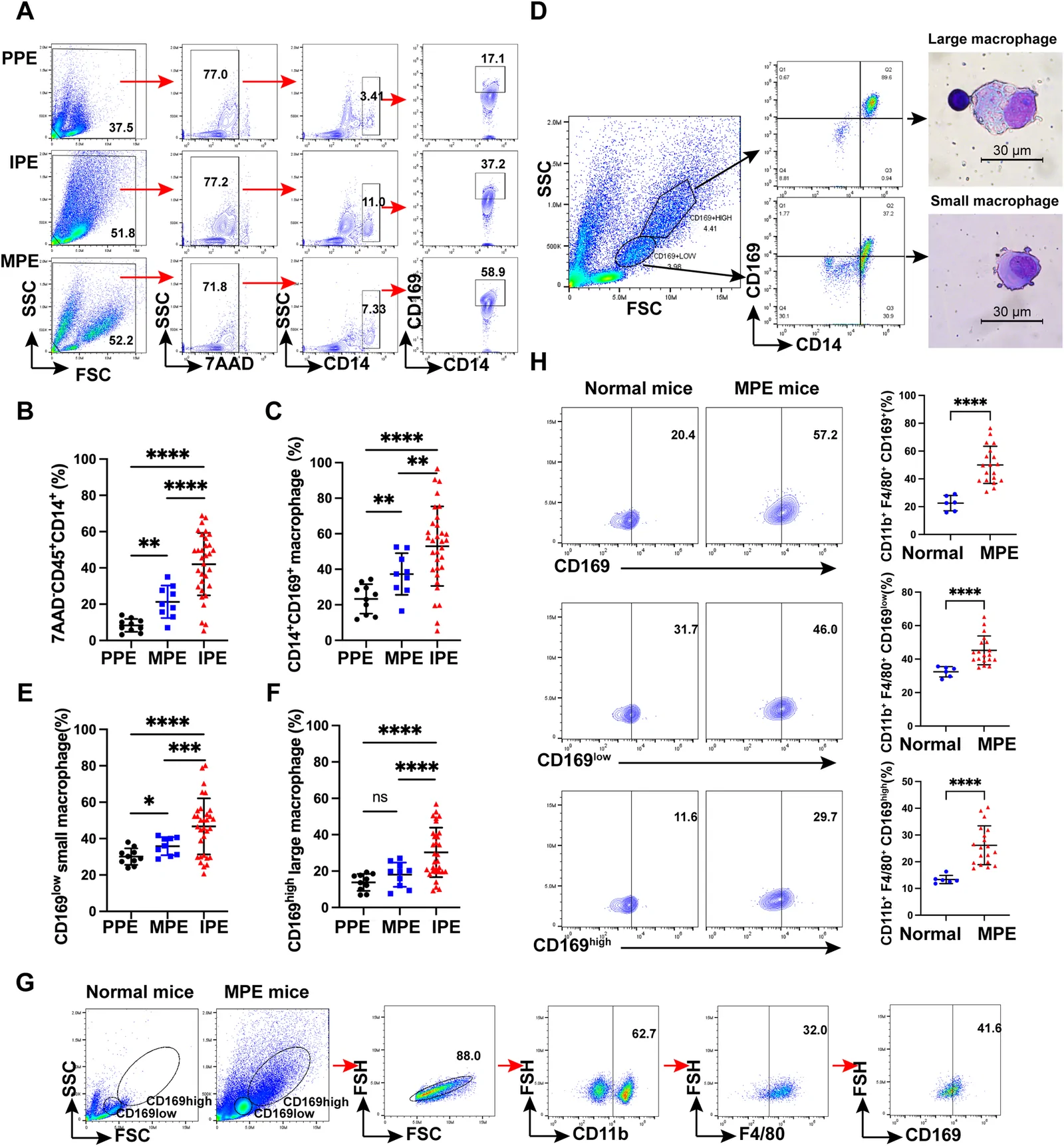
Increased infiltration of CD169^+^ macrophages in MPE. **(A)** Flow Cytometry Gating Strategy for PPE, IPE, and MPE. **(B)** Comparison of CD45^+^CD14^+^ Macrophage Proportions in PPE, IPE, and MPE Patients. **(C)** Comparison of CD14^+^CD169^+^ Macrophage Proportions in PPE, IPE, and MPE Patients. **(D)** Flow Cytometry Gating Strategy for CD169^low^ Small Macrophages and CD169^high^ Large Macrophages in PPE, IPE, and MPE Patients. **(E)** Comparison of CD169^low^ Small Macrophage Expression in PPE, IPE, and MPE Patients. **(F)** Comparison of CD169^high^ Large Macrophage Expression in PPE, IPE, and MPE Patients. **(G)** Flow Cytometry Gating Strategy for CD169^+^ Macrophage Expression in Normal Mice and MPE Mice. **(H)** Representative Flow Cytometry Plots and Comparison of CD169^+^ Macrophages, CD169^low^ Small Macrophages, and CD169^high^ Large Macrophages in Normal Mice and MPE Mice. Normal Mice *n* = 6, MPE Mice *n* = 19. Data are presented as mean ± SEM. ns *P* > 0.05, * *P* < 0.05, ** *P* < 0.01, *** *P* < 0.001, **** *P* < 0.0001. PPE, Postoperative pleural effusion, *n* = 10. IPE, Inflammatory pleural effusion, *n* = 10. MPE, Malignant pleural effusion, *n* = 32.

To characterize the macrophage landscape in MPE, we performed flow cytometric analysis of PPE from stage IA lung adenocarcinoma patients, IPE from pneumonia patients, and MPE from lung Adenocarcinoma patients. The gating strategy is shown in Figure 1A, and the patient information is shown in Table 1. Compared with PPE and IPE controls, MPE samples exhibited a prominent expansion of the CD45⁺CD14⁺ macrophage compartment (MPE vs. PPE: 42.05% ± 17.21% vs*.* 21.32% ± 8.94%, *P* < 0.0001; MPE vs. IPE: 42.05% ± 17.21% vs. 8.33% ± 3.58%, *P* < 0.0001; Figure 1B). Furthermore, the proportion of CD45⁺CD14⁺CD169⁺ macrophages was significantly increased in MPE compared with both PPE and IPE samples (MPE vs. PPE: 52.98% ± 22.36% vs. 37.31% ± 11.72%, *P* < 0.01; MPE vs. IPE: 52.98% ± 22.36% vs. 23.33% ± 8.31%, *P* < 0.0001; Figure 1C). To further characterize this CD169⁺ macrophage population, we performed additional phenotypic stratification based on cell size and granularity, together with CD169 fluorescence intensity. This analysis identified two distinct CD169⁺ macrophage subsets: CD169^low^ small macrophages and CD169^high^ large macrophages (Figure 1D). Importantly, both CD169⁺ macrophage subsets were preferentially accumulated in MPE. The frequency of CD169^low^ small macrophages was significantly increased in MPE compared with PPE and IPE samples (MPE *vs.* PPE: 46.71% ± 15.42% vs. 30.12% ± 4.54%, *P* < 0.0001; MPE *vs.* IPE: 46.71% ± 15.42% vs*.* 35.82% ± 4.94%, *P* < 0.001; Figure 1E). Similarly, CD169^high^ large macrophages showed a marked expansion in MPE compared with PPE and IPE controls (MPE vs. PPE: 30.27% ± 13.59% vs. 13.82% ± 4.66%, *P* < 0.0001; MPE vs. IPE: 30.27% ± 13.59% vs. 18.03% ± 6.64%, *P* < 0.0001; Figure 1F).

**Table 1:** Baseline clinical characteristics of patients with three types of pleural effusion.

Variable	Postoperative pleural effusion in stage IA lung adenocarcinoma	inflammatory pleural effusion	Malignant pleural effusion derived from lung adenocarcinoma	*P* value
Number of cases	10	10	32	
Age (years)				*P* = 0.828
Mean ± SD	69.45 ± 7.50	70.62 ± 9.66	72.50 ± 9.30	
Gender				*P* = 0.949
Male	7	7	21	
Female	3	3	11	
Pathological type	Lung adenocarcinoma	_	Lung adenocarcinoma	
Tumor stage	Stage IA	_	Stage Ⅳ	
Subsequent therapeutic regimens	_	_	Platinum-based chemotherapy	

These results indicate that the proportion of CD169^+^ macrophages is significantly increased in malignant pleural effusion of human lung cancer, with both CD169^low^ small macrophages and CD169^high^ large macrophages showing markedly elevated infiltration.

### 3.2 Increased infiltration of CD169^+^ macrophages in MPE in a mouse model of lung adenocarcinoma

Next, flow cytometric analysis was performed on pleural lavage fluid from normal mice and MPE from MPE mice (Figure 1H). The results revealed that the proportions of CD169^+^ macrophages, CD169^low^ small macrophages, and CD169^high^ large macrophages were also significantly higher in MPE mice compared to those in the pleural lavage fluid of normal mice (CD169^+^ macrophages, 50.07% ± 13.39% vs. 22.53% ± 5.48%, *P* < 0.0001; CD169^low^ small macrophages, 45.23% ± 8.61% vs. 32.43% ± 3.06%, *P* < 0.0001; CD169^high^ large macrophages: 26.15% ± 7.28% vs. 13.35% ± 1.56%, *P* < 0.0001, Figure 1H).

### 3.3 CD169^high^ large macrophages in MPE of human lung adenocarcinoma exhibit M2-like immunosuppressive phenotype

**Figure 2:**
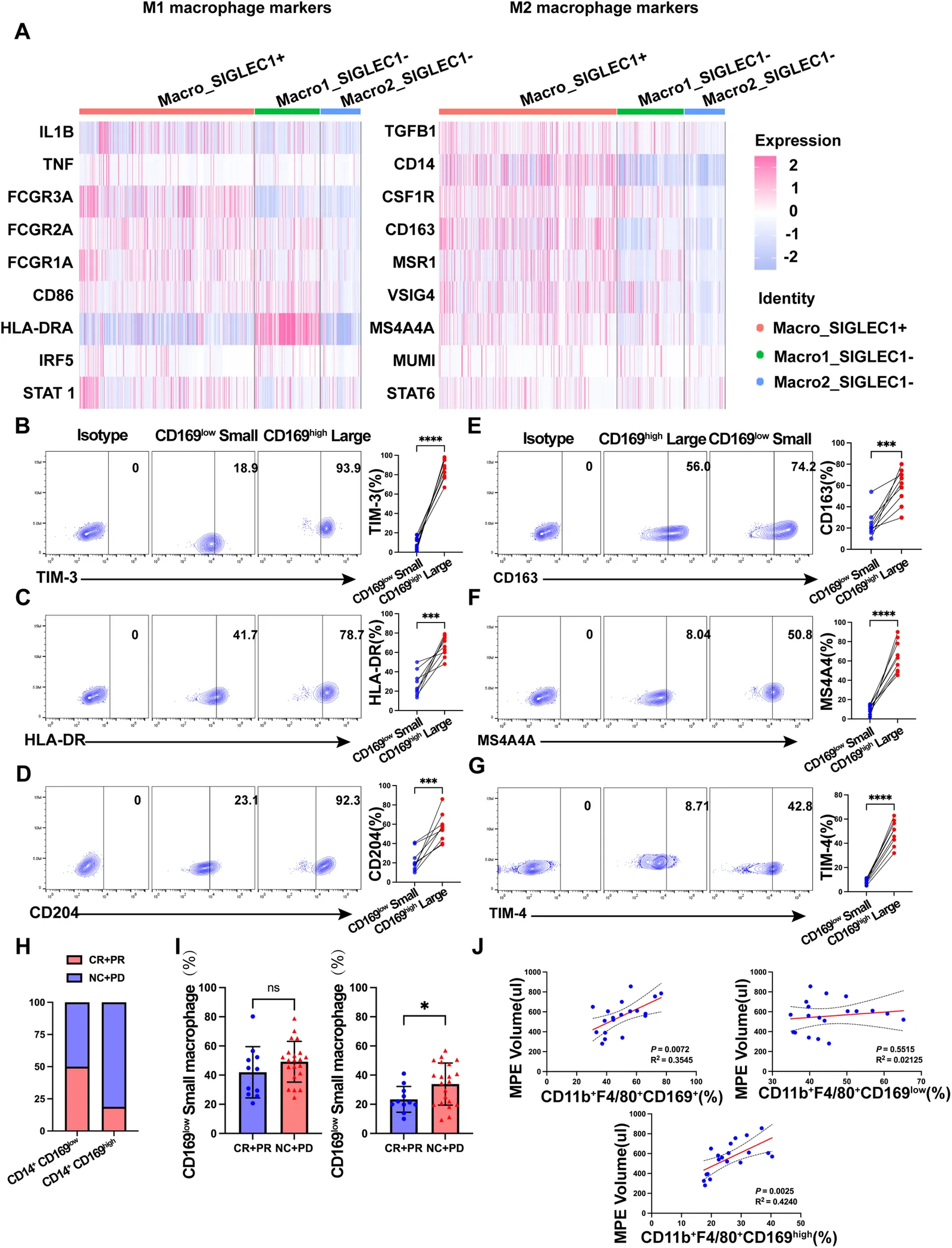
CD169^high^ large macrophages in MPE from human lung adenocarcinoma exhibit an immunosuppressive phenotype and are associated with poor prognosis. **(A)** Expression of M1 and M2 macrophage molecular markers on CD169^+^ macrophages. The color indicates average expression level. **(B)**–**(G)** Expressions of TIM-3, HLA-DR, CD204, MS4A4A, CD163, and TIM-4 on CD169^low^ small macrophages and CD169^high^ large macrophages, including representative flow cytometry plots and statistical graphs. **(H)** Effective control rate of pleural effusion in the high- and low-CD169 expression groups. **(I)** Comparison of CD169^low^ small macrophages and CD169^high^ large macrophages abundances between the pleural effusion effective and ineffective control groups. **(J)** Correlation analysis of CD169^+^ macrophages, CD169^low^ small macrophages and CD169^high^ large macrophages abundances with MPE volume in murine MPE. Data are presented as mean ± SEM. ns *P* > 0.05, * *P* < 0.05, ** *P* < 0.01, *** *P* < 0.001, **** *P* < 0.0001. MPE, Malignant pleural effusion. CR, Complete response. PR, Partial response. NC, Unchanged. PD, Progressive disease.

To further explore the biological functions of CD169^+^ macrophages, single-cell sequencing data from the GSE131907 dataset were analyzed to characterize the expression of canonical M1 and M2 macrophage markers in CD169^+^ macrophages. Higher expression levels of M2 macrophage markers were observed in CD169^+^ macrophages (Figure 2A). Subsequently, flow cytometry was performed to validate the phenotypic characteristics of CD169^+^ macrophages in MPE samples from patients with lung adenocarcinoma. The results showed that the expressions of TIM-3, HLA-DR, CD204, MS4A4A, CD163, and TIM-4 on CD169^high^ large macrophages were significantly higher than those on CD169^low^ small macrophages (*P* < 0.05, Figures 2B–G), suggesting that CD169^high^ large macrophages in MPE of human lung adenocarcinoma exhibit an immunosuppressive phenotype.

### 3.4 CD169^high^ large macrophages in MPE of lung adenocarcinoma are associated with poor prognosis

Next, to investigate whether the increased expression of CD169^+^ macrophages in MPE has an impact on patients' prognosis, we evaluated the relationship between the CD169 expression level in MPE patients and the effective control rate of pleural effusion. The results showed that the effective control rate of pleural effusion in the high-CD169 expression group of MPE patients was significantly lower than that in the low-CD169 expression group (14.3% vs. 51.9%, Figure 2H).

According to the control status of pleural effusion, MPE patients were divided into an effective control group and an ineffective control group. The expression of CD169^+^ macrophages was detected. There was no significant difference in the proportion of CD169^low^ small macrophages between the ineffective control group and the effective control group (46.40% ± 13.37% vs. 44.74% ± 16.37%, *P* > 0.05, Figure 2I). However, the proportion of CD169^high^ large macrophages in the ineffective control group was significantly higher than that in the effective control group (35.01% ± 13.13% vs. 24.29% ± 9.71%, *P* < 0.01, Figure 2I).

In addition, a correlation analysis was performed between the proportions of CD169^+^ macrophages, CD169^low^ small macrophages, and CD169^high^ large macrophages in mouse MPE and the volume of mouse MPE. It was found that the proportion of CD169^+^ macrophages in mouse MPE from lung cancer was positively correlated with the volume of MPE. There was no correlation between the proportion of CD169^low^ small macrophages in mouse MPE from lung cancer and the volume of MPE. The proportion of CD169^high^ large macrophages in mouse MPE from lung cancer was positively correlated with the volume of MPE (Figure 2J).

The above results indicate that CD169^high^ large macrophages in human lung cancer associated MPE are negatively correlated with the control rate, and CD169^high^ large macrophages in mouse lung cancer associated MPE are positively correlated with the volume of mouse MPE. These findings suggest that CD169^high^ large macrophages are involved in the progression of MPE.

### 3.5 Increased secretion of CXCL2 and CXCL8 in MPE of human lung adenocarcinoma

**Figure 3:**
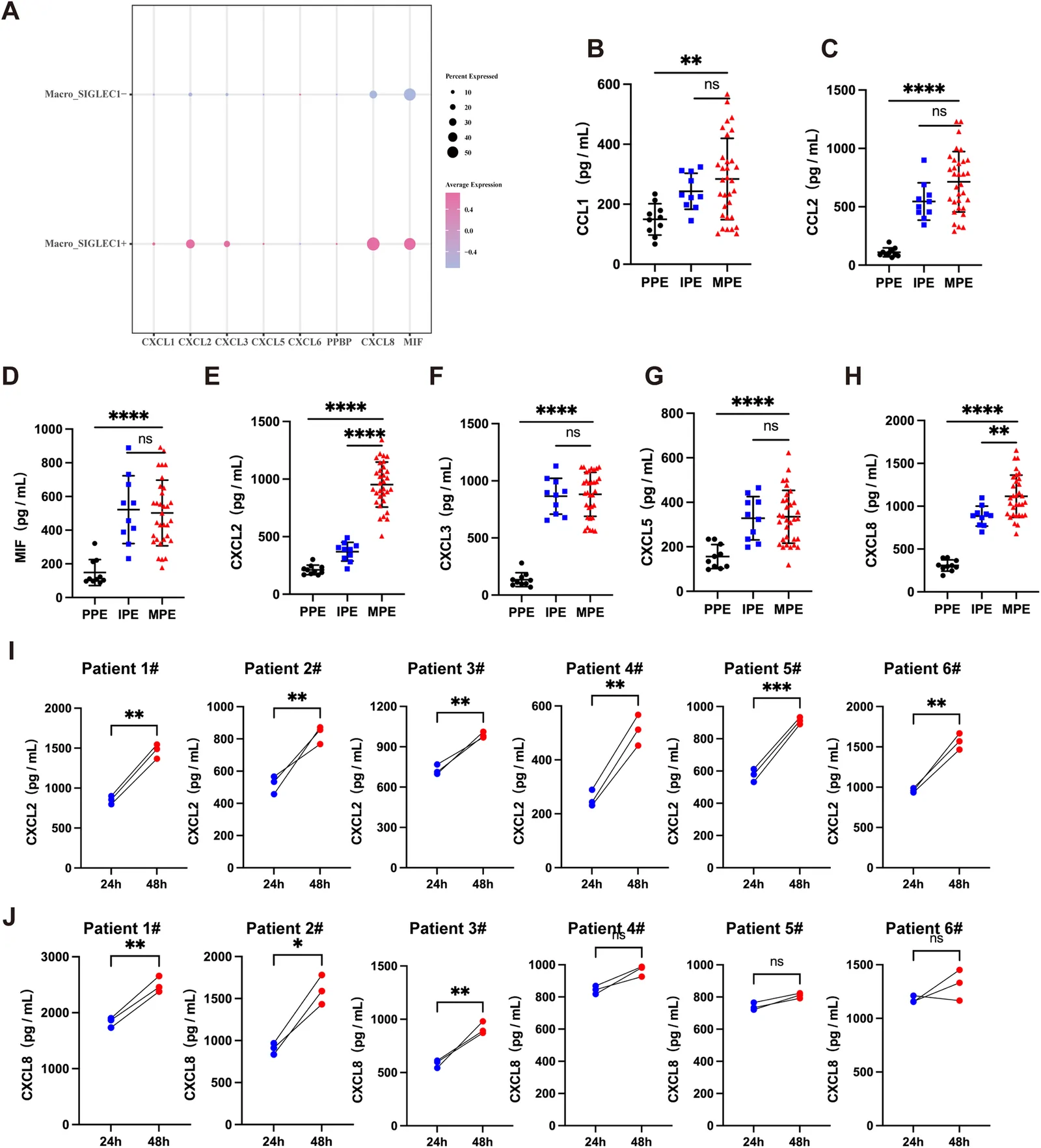
Comparison of chemokine expression in PPE, IPE, and MPE. **(A)** Chemokines and receptors Expression on CD169^+^ macrophages. Dot size reflects the proportion of cells expressing a given gene, and color indicates average expression level. **(B)**–**(H)** Expression of CCL1,CCL2,MIF,CXCL2,CXCL3,CXCL5,CXCL8 in PPE, IPE, and MPE. **(I)** Changes in the expression of CXCL2 on CD14^+^CD169^+^ macrophages in MPE of lung cancer patients at 24 h and 48 h. **(J)** Changes in the expression of CXCL8 on CD14^+^CD169^+^ macrophages in MPE of lung cancer at 24 h and 48 h. Data are presented as mean ± SEM. ns *P* > 0.05, * *P* < 0.05, ** *P* < 0.01, *** *P* < 0.001, **** *P* < 0.0001. PPE, Postoperative pleural effusion, *n* = 10. IPE, Inflammatory pleural effusion, *n* = 10. MPE, Malignant pleural effusion, *n* = 32.

Previous studies have shown that the chemokine family can regulate macrophage activation, polarization, and changes in their molecular phenotypes (11). We analyzed several chemokines that act on macrophages using single-cell sequencing data from the GSE131907 dataset (12–15) (Figure 3A). Then we used the ELISA method to verify the analysis results. The results showed that the concentration of CXCL2 in the MPE of lung cancer was significantly higher than that in IPE (932.1 pg/mL ± 201.5 pg/mL vs. 369.5 pg/mL ± 80.9 pg/mL, *P* < 0.0001, Figure 3E). The concentration of CXCL8 in the MPE of lung cancer was also higher than that in IPE, but the difference was not as significant as that of CXCL2 (1,084 pg/mL ± 272.1 pg/mL vs. 884.2 pg/mL ± 116.1 pg/mL, *P* < 0.05, Figure 3H).

Next, we sorted CD14^+^CD169^+^ macrophages from the MPE of 6 lung cancer patients and cultured them *in vitro*. The supernatants were collected at 24 h and 48 h respectively to detect the concentrations of CXCL2 and CXCL8. The results showed that over time, compared with CXCL8, the secretion of CXCL2 by MPE CD14^+^CD169^+^ macrophages cultured *in vitro* was more active during the 24–48 h period (Figures 3I,J). These results suggest that CD14^+^CD169^+^ macrophages in the MPE of lung cancer can produce CXCL2, and the increased infiltration of CD169^high^ large macrophages in MPE may be mainly influenced by CXCL2.

### 3.6 CXCL2/CXCR2 pathway regulates the phenotype of CD169^high^ large macrophages

**Figure 4:**
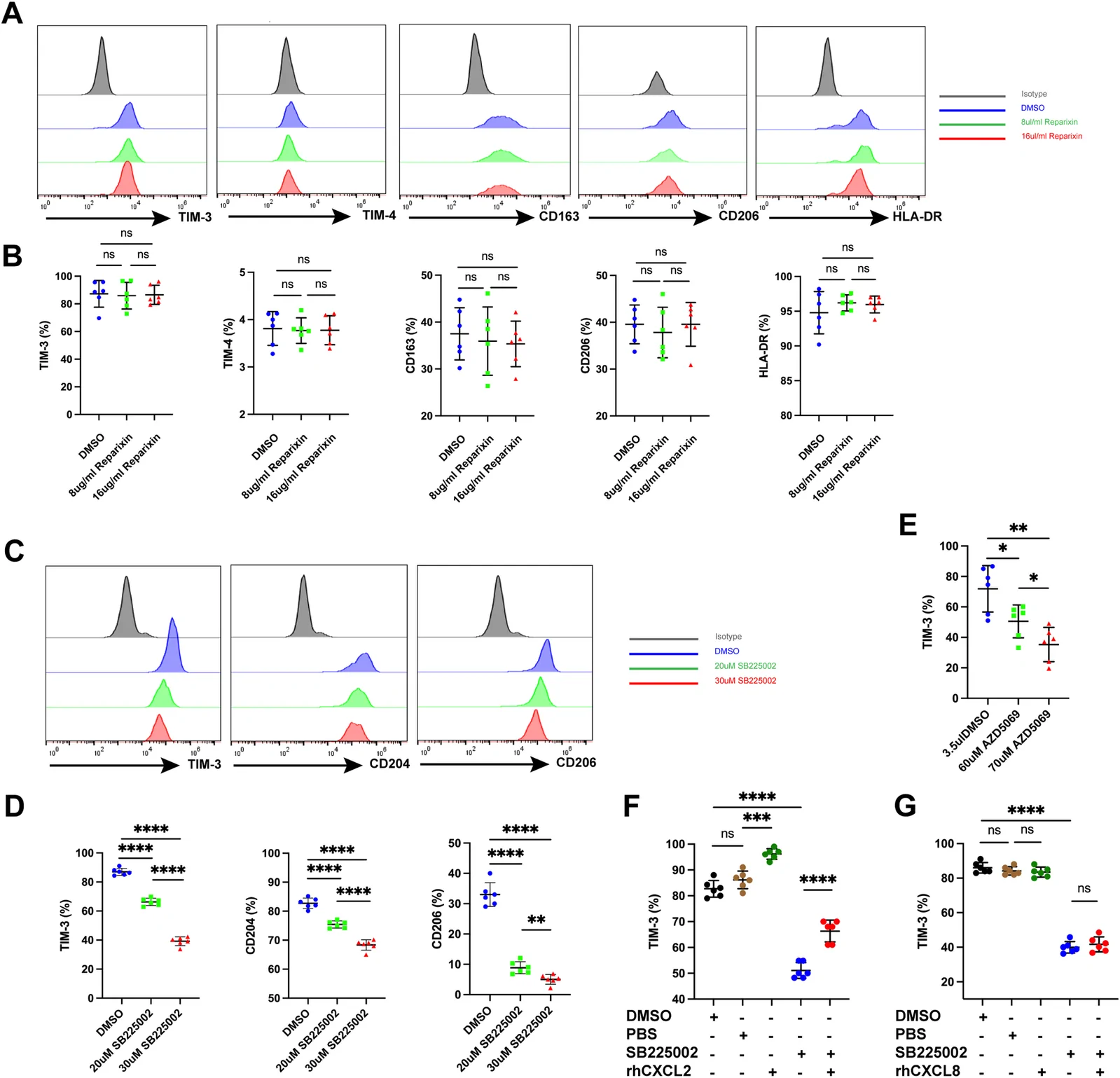
CXCL2/CXCR2 pathway regulates the phenotype of CD169^high^ large macrophages. **(A)** Representative flow cytometry histograms showing the changes in the expression of relevant molecules on the surface of CD169^high^ large macrophages after the addition of Reparixin. **(B)** Statistical graphs depicting the percentages of the expression of relevant molecules on the surface of CD169^high^ large macrophages after the addition of Reparixin. **(C)** Representative flow cytometry histograms showing the changes in the expression of relevant molecules on the surface of CD169^high^ large macrophages after the addition of SB225002. **(D)** Statistical graphs depicting the percentages of the expression of relevant molecules on the surface of CD169^high^ large macrophages after the addition of SB225002. **(E)** Statistical graphs depicting the percentages of the expression of TIM-3 on the surface of CD169^high^ large macrophages after the addition of AZD5069. **(F)** Statistical graphs depicting the percentages of the expression of TIM-3 on the surface of CD169^high^ large macrophages after the addition of SB225002 and rhCXCL2. **(G)** Statistical graphs depicting the percentages of the expression of TIM-3 on the surface of CD169^high^ large macrophages after the addition of SB225002 and rhCXCL8. Data are presented as mean ± SEM. ns *P* > 0.05, * *P* < 0.05, ** *P* < 0.01, *** *P* < 0.001, **** *P* < 0.0001.

Therefore, we sorted CD14^+^CD169^+^ macrophages from the MPE of lung cancer patients and added Reparixin, an inhibitor of CXCR1. After *in vitro* culture for 48 h, we used flow cytometry to detect the relevant molecular phenotypes on CD169^high^ large macrophages. We found that the addition of Reparixin did not affect the phenotype of CD169^high^ large macrophages (Figures 4A,B). Next, we sorted CD14^+^CD169^+^ macrophages from the MPE of lung cancer patients and treated them with SB225002, an inhibitor of CXCR2, for 48 h. Then we detected the expression of phenotypic molecules on the cells. The results showed that, compared with the DMSO group, the expression of immunosuppressive molecules TIM-3, CD204, and CD206 on CD169^high^ large macrophages in the SB225002 group was decreased. Moreover, with the increase of the drug—administration concentration, the expression of TIM-3, CD204, and CD206 further decreased (*P* < 0.05, Figures 4C,D). In addition, when we added another CXCR2 inhibitor, AZD5069, the expression of the immune checkpoint molecule TIM-3 on CD169^high^ large macrophages also decreased with the increase in the drug—administration concentration (*P* < 0.05, Figure 4E). Next, rhCXCL2 and rhCXCL8 were added to the CD14^+^CD169^+^ macrophages that had been treated with SB225002. It was found that the expression of TIM-3 increased again after the addition of rhCXCL2 (*P* < 0.05, Figure 4F), whereas no change was observed after the addition of rhCXCL8 (Figure 4G). CXCL8 can bind to both CXCR1 and CXCR2, but has different affinities for CXCR1 and CXCR2, with a significantly higher affinity for CXCR1 (16). This can explain why the phenotype of CD169 macrophages did not change after adding CXCR2 inhibitors followed by CXCL8. These findings suggest that CXCL2/CXCR2 pathway regulates the phenotype of CD169^high^ large macrophages.

**Figure 5:**
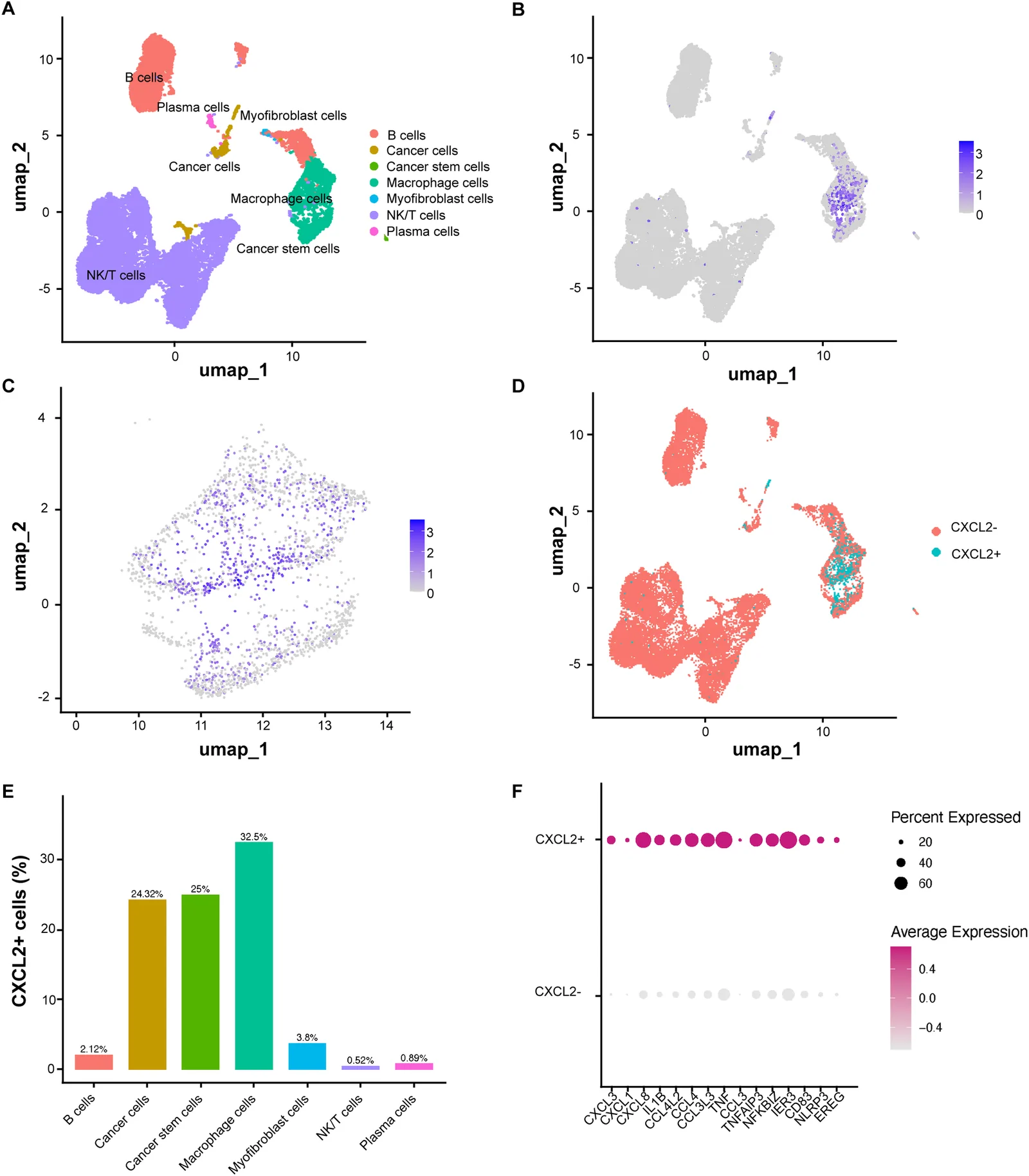
Single-cell transcriptomic analysis of cellular composition and CXCL2 expression profiles in MPE samples. **(A)** UMAP plot of unsupervised clustering and cell type annotation for all cells in the MPE sample. Cell populations were identified based on characteristic marker genes, revealing seven major cell subpopulations: B cells, cancer cells, cancer stem cells, macrophages, myofibroblasts, NKT cells, and plasma cells. **(B)** UMAP plot showing CXCL2 expression patterns across all cells in the MPE sample. The color gradient indicates relative levels of CXCL2 expression. **(C)** UMAP plot depicting CXCL2 expression patterns within the macrophage subpopulation, illustrating the distribution of CXCL2 expression among macrophages. **(D)** UMAP plot classifying cells based on CXCL2 expression status. Cells are divided into CXCL2-negative (CXCL2⁻, red) and CXCL2-positive (CXCL2⁺, green) groups according to their CXCL2 expression. **(E)** Bar chart showing the proportion of CXCL2⁺ cells across different cell subpopulations. The *y*-axis represents the percentage of CXCL2⁺ cells within each cell type. **(F)** Dot plot of differentially expressed genes between CXCL2⁺ and CXCL2⁻ macrophages. The *x*-axis represents individual genes, while the *y*-axis shows the two macrophage groups. Dot size reflects the proportion of cells expressing a given gene, and color indicates average expression level.

To identify the potential cellular source of CXCL2 in the MPE microenvironment, we reanalyzed the publicly available single-cell RNA-sequencing dataset and examined CXCL2 expression across the major cell populations. The dataset comprised seven major cellular populations, including B cells, cancer cells, cancer stem cells, macrophages, myofibroblasts, NK/T cells, and plasma cells (Figure 5A). CXCL2 expression was predominantly localized to the macrophage compartment, with a marked enrichment of CXCL2-expressing cells within the macrophage cluster (Figures 5B–D). Quantitatively, 32.5% of macrophages expressed CXCL2, representing the highest proportion among the analyzed cell populations, followed by cancer stem cells (25.0%) and cancer cells (24.32%). In contrast, CXCL2 expression was detected in only a small fraction of B cells (2.12%), myofibroblasts (3.8%), NKT cells (0.52%), and plasma cells (0.89%) (Figure 5E). These findings identify macrophages as the predominant CXCL2-expressing population at the transcript level, while also indicating that malignant and cancer stem-like cells may contribute to the CXCL2 pool within the MPE microenvironment.

### 3.7 SB225002 inhibits the formation of MPE in mice

**Figure 6:**
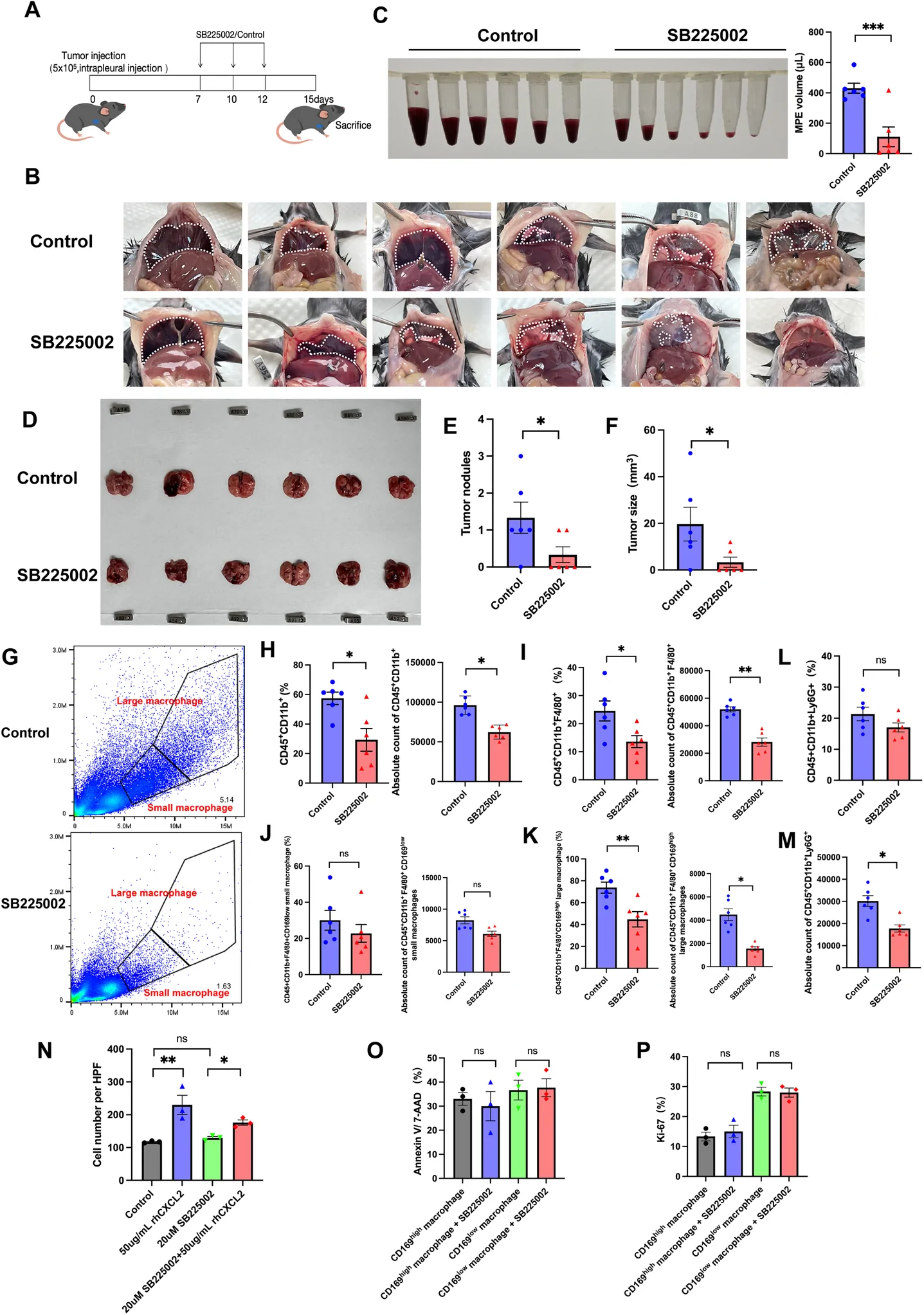
Effects of SB225002 on the formation of MPE. **(A)** Scheduled timeline for MPE mouse model establishment and drug administration. The model was established on day 0, and on days 7, 10, and 12, the mice were treated with SB225002 or the vehicle control. Finally, the mice were sacrificed on day 15. **(B)** *in vivo* images of MPE in mice from the control and experimental groups (dotted lines indicate the range of pleural effusion), *n* = 6. **(C)** *ex vivo* images and statistical histogram of MPE in mice from the control and experimental groups. **(D)** Lung images of MPE mice after dissection from the control and experimental groups, *n* = 6. **(E)** Statistical histograms of tumor nodules in the thoracic cavities of MPE mice from the control and experimental groups, *n* = 6. **(F)** Statistical histograms of tumor size in the thoracic cavities of MPE mice from the control and experimental groups, *n* = 6. **(G)** Representative flow cytometry plots illustrating the subpopulations of CD169^+^ macrophages in MPE derived from control and experimental mice. **(H)** Quantitative comparison of the proportion and absolute number of myeloid cells in MPE between the control group and the experimental group, *n* = 6. **(I)** Quantitative comparison of the proportion and absolute number of macrophages in MPE between the control group and the experimental group, *n* = 6. **(J)** Quantitative comparison of the proportion and absolute number of CD169^low^ small macrophages in MPE between the control group and the experimental group, *n* = 6. **(K)** Quantitative comparison of the proportion and absolute number of CD169^high^ large macrophages in MPE between the control group and the experimental group, *n* = 6. **(L)** Quantitative comparison of the proportion of neutrophil in MPE between the control group and the experimental group, *n* = 6. **(M)** Quantitative comparison of the absolute number of neutrophil in MPE between the control group and the experimental group, *n* = 6. **(N)** Quantification of transwell assay. PBMCs were seeded into the upper chambers of Transwell inserts with 8-μm pore membranes in serum-free medium. rhCXCL2 was added to the lower chambers, SB225002 was added to the upper chambers, count the cells in the lower chamber after incubation for 4 h. **(O)** Flow-cytometric analysis of Annexin V/7-AAD in CD169^high^ and CD169^low^ macrophage following treatment with SB225002 or vehicle control for 24 h. **(P)** Flow-cytometric analysis of intracellular Ki-67 expression in CD169^high^ and CD169^low^ macrophage populations following treatment with SB225002 or vehicle control for 24 h. Data are presented as mean ± SEM. ns *P* > 0.05, * *P* < 0.05, ** *P* < 0.01, *** *P* < 0.001, **** *P* < 0.0001. MPE, Malignant pleural effusion.

To further verify the effect of SB225002 *in vivo*. In this experiment, a mouse model of MPE was established on day 0. Then, on days 7, 10, and 12, the mice were treated with SB225002 or the vehicle control, respectively. Finally, the mice were sacrificed on day 15 (Figure 6A). The results showed that the volume of MPE in the experimental group of mice treated with SB225002 was significantly less than that in the control group (Figures 6B,C). Besides, we additionally quantified macroscopic tumor burden in the murine MPE model. Compared with vehicle-treated mice, SB225002-treated mice exhibited a significant reduction in tumor burden (Figures 6D–F). These findings indicate that the effect of CXCR2 inhibition was not limited to the regulation of pleural fluid accumulation, but was also associated with reduced tumor burden in the MPE model. Flow cytometry analysis of the MPE from the two groups of mice revealed that the number of CD169^+^ macrophages in the MPE of the experimental group was significantly reduced compared with that in the control group, especially the CD169^high^ large macrophage population (Figure 6G).

Next, in order to characterize the effect of CXCR2 inhibition on the pleural myeloid compartment, we analyzed flow-cytometric data from MPE mice treated with vehicle or SB225002. SB225002 treatment significantly reduced both the proportion and absolute number of CD45^+^CD11b^+^ myeloid cells and total macrophages. Notably, CD169^high^ macrophages were significantly decreased in both frequency and absolute number, whereas CD169^low^ macrophages were not significantly affected. In addition, SB225002 reduced the absolute number of neutrophils, although their relative frequency among CD45^+^CD11b^+^ cells remained unchanged (Figures 6H–M). These findings indicate that CXCR2 inhibition induces broader changes in the pleural myeloid compartment rather than selectively targeting CD169^high^ macrophages, but CD169^high^ macrophages still represent an important cellular population associated with MPE progression.

In order to further explain the mechanisms underlying the reduction of CD169^high^ macrophages following CXCR2 inhibition, we examined macrophage migration, proliferation, and apoptosis. We first performed an *in vitro* Transwell migration assay to determine whether CXCL2/CXCR2 signaling directly regulates macrophage chemotaxis. Recombinant human CXCL2 significantly increased macrophage migration, whereas pharmacological inhibition of CXCR2 with SB225002 significantly reduced macrophage migration (Figure 6N). These findings indicate that CXCL2 promotes macrophage migration and support a role for CXCR2 signaling in macrophage recruitment. We next examined macrophage apoptosis using Annexin V/7-AAD staining. No significant differences in apoptotic cells were observed between the control and SB225002-treated groups in either the CD169^high^ or CD169^low^ macrophage populations (Figure 6O). Collectively, these findings suggest that the CXCL2/CXCR2 axis contributes primarily to macrophage recruitment rather than substantially affecting macrophage proliferation or apoptosis. We further evaluated whether CXCR2 inhibition altered macrophage proliferative activity. Intracellular Ki-67 staining revealed no significant differences in the proportion of Ki-67-positive cells within either the CD169^high^ or CD169^low^ macrophage populations following SB225002 treatment (Figure 6P). These results suggest that altered macrophage proliferation is unlikely to account for the reduction in CD169^high^ macrophages following CXCR2 inhibition. Collectively, these findings suggest that the CXCL2/CXCR2 axis contributes primarily to macrophage recruitment rather than substantially affecting macrophage proliferation or apoptosis.

## 4 Discussion

In this study, we profiled the proportions and phenotypic molecule expression of CD169^+^ macrophages in human clinical specimens and murine MPE models. We identified a novel immunosuppressive cell subset, CD169^high^ large macrophages, which is significantly enriched in lung adenocarcinoma-derived MPE and closely correlated with poor clinical control of MPE. Furthermore, we first demonstrated that the CXCL2/CXCR2 signaling pathway sustains the immunosuppressive phenotype of CD169^high^ large macrophages, thereby facilitating MPE progression. These findings provide novel mechanistic insights into the immune regulatory network of MPE and offer a potential precision therapeutic target based on macrophage subsets for MPE treatment.

CD169, also termed sialoadhesin or Siglec-1, is selectively expressed on specific macrophage populations and primarily participates in phagocytosis, antigen presentation, and immune response modulation (17, 18). Accumulating evidence has highlighted the critical involvement of CD169^+^ macrophages in tumorigenesis and cancer progression. In lung cancer, CD169^+^ macrophages drive tumor progression by inducing epithelial-mesenchymal transition, promoting tumor cell proliferation and invasion, and enhancing regulatory T cell (Treg)-mediated suppression of CD8^+^ T cell cytotoxicity (19). In breast cancer, tumor cell-derived inflammatory factors upregulate CD169 expression in tumor-associated macrophages (TAMs), which subsequently induces CCL8 secretion to further amplify TAM polarization via a positive feedback loop. Notably, elevated CD169 and CCL8 expression correlates with unfavorable prognosis in breast cancer patients (20). In melanoma, CD169^+^ macrophages in regional lymph nodes bind to circulating tumor cells, promote lymph node colonization, and accelerate melanoma progression (17).

Notably, CD169^+^ macrophages exert context-dependent and even opposing functions across different cancer types. In liver cancer, CD169 serves as a co-stimulatory molecule to trigger CD8^+^ T cell activation and immune responses (21). Moreover, CD169^+^ macrophages mediate cross-presentation of apoptotic tumor antigens to CD8^+^ T cells, thereby promoting T cell proliferation and the secretion of anti-tumor effector molecules, including IL-2, IFN-γ, and granzyme B (22). In colorectal cancer, CD169^+^ macrophages engage the CD169-CD43 axis to activate CD8^+^ T cells in regional lymph nodes and elicit anti-tumor immunity (23). In glioblastoma, CD169^+^ macrophages possess enhanced phagocytic capacity and boost anti-tumor immune responses by recruiting activated T cells into the tumor microenvironment (8). Consistently, high infiltration of CD169^+^ macrophages predicts favorable clinical outcomes in gastric cancer (24). Similarly, CD169^+^ macrophages in tumor-draining lymph nodes strengthen anti-tumor immunity by expanding CD8^+^ T cell populations, and high CD169 expression represents a robust favorable prognostic biomarker in bladder cancer (25).

The dual roles of CD169^+^ macrophages in tumor progression are highly dependent on the heterogeneous tissue microenvironments of different malignancies. Distinct microenvironmental cues shape the polarization and functional status of CD169^+^ macrophages, resulting in divergent pro- or anti-tumor phenotypes. This context dependency explains why CD169^+^ macrophages promote disease progression in MPE, lung cancer, breast cancer, and melanoma, whereas they exert tumor-suppressive effects in liver cancer, colorectal cancer, glioblastoma, gastric cancer, and bladder cancer. Nevertheless, the precise mechanisms by which CD169^+^ macrophages regulate immune responses and the upstream signaling cascades governing their functional heterogeneity across cancer types remain poorly characterized.

Classically, CD169 (Siglec-1) is recognized as an interferon-inducible molecule, and IFN-γ is well documented to drive CD169 expression on macrophages and promote a pro-inflammatory, antitumor phenotype under infectious and certain tumor contexts (8, 26). For instance, CD169^+^ macrophages induced by NK cell-derived IFN-*γ* exert potent antitumor effects in glioblastoma by enhancing T cell recruitment and phagocytic function (8). However, the CD169^high^ large macrophage subset identified in our study displayed a dominant immunosuppressive phenotype in lung adenocarcinoma-associated MPE, which is phenotypically and functionally distinct from the IFN-γ induced pro-inflammatory CD169^+^ macrophages. This divergence highlights the context-dependent plasticity of CD169^+^ macrophage polarization. Furthermore, the inhibitory effect of IFN-*γ* on the ELR^+^ CXC chemokine–CXCR2 axis provides an important upstream regulatory perspective for our findings. As a shared receptor for CXCL2 and CXCL8, CXCR2-mediated myeloid cell trafficking and functional polarization can be negatively regulated by IFN-γ. Previous studies have demonstrated that IFN-γ suppresses the expression and secretion of CXCL8 in pancreatic cancer cells, thereby blocking CXCR2-dependent recruitment of immunosuppressive macrophages and enhancing the efficacy of anti-PD-1 immunotherapy (27, 28). Given the high structural and functional homology between CXCL2 and CXCL8, this inhibitory regulation can be reasonably extrapolated to the CXCL2/CXCR2 pathway investigated in the present study. In line with this notion, clinical evidence has shown that IFN-*γ* levels are significantly reduced in malignant pleural effusions compared with inflammatory pleural effusions, and effector T cells in MPE exhibit impaired IFN-*γ* production capacity (9, 29). We therefore hypothesize that insufficient IFN-γ in the MPE microenvironment, partially resulting from T cell exhaustion, relieves the endogenous suppression of the CXCL2/CXCR2 signaling axis. This disinhibition in turn promotes the accumulation of CD169^high^ large macrophages, ultimately contributing to MPE progression.

Several limitations of this study should be acknowledged. First, we did not directly investigate the role of IFN-*γ* in regulating CD169^high^ large macrophages, nor did we verify the crosstalk between IFN-*γ* signaling and the CXCL2/CXCR2 pathway in MPE. Future studies will include *in vitro* IFN-*γ* stimulation assays on primary CD169^high^ macrophages isolated from clinical MPE samples, *in vivo* IFN-*γ* intervention in the murine MPE model, and detection of IFN-*γ* concentrations in pleural effusion specimens, to systematically elucidate the IFN-*γ*–CXCL2/CXCR2–CD169^high^ macrophage regulatory axis.

Dysregulated chemokine signaling is widely recognized as a critical driver of tumor pathogenesis (30). Numerous chemokine ligand–receptor pairs exert versatile pro- or anti-tumor effects by modulating tumor angiogenesis, cell proliferation, differentiation, immune evasion, and metastasis (31). CXCR2, a receptor for seven CXC chemokines (CXCL1, CXCL2, CXCL3, CXCL5, CXCL6, CXCL7, and CXCL8) (32), plays a predominant pro-tumor role in multiple cancers. In patients with lung adenocarcinoma and squamous cell carcinoma, CXCR2 expression is upregulated in both tumor cells and stromal cells, and high CXCR2 levels correlate with reduced overall survival. Consistently, murine lung tumor tissues exhibit elevated expression of CXCL1, CXCL2, and CXCL5 compared with normal lung tissues. Pharmacological inhibition of CXCR2 by SB225002 induces tumor cell apoptosis and senescence, reverses epithelial-mesenchymal transition, and impairs tumor cell proliferation *in vitro*. *In vivo* CXCR2 blockade suppresses tumor growth, reduces intratumoral neutrophil infiltration, enhances CD8^+^ T cell activation, and improves cisplatin sensitivity in lung cancer models (10).

In breast cancer, triple-negative breast cancer, the most aggressive subtype, exhibits higher expression of CXCR2 ligands (CXCL1, CXCL2, CXCL5, and CXCL8) than other breast cancer subtypes. CXCR2 is predominantly expressed by tumor-infiltrating neutrophils and is associated with advanced, invasive breast cancer phenotypes (33, 34). In colorectal cancer, elevated CXCR2 expression serves as an independent adverse prognostic factor for overall and disease-free survival (35). In melanoma, CXCR2 inhibition suppresses tumor growth and augments the therapeutic efficacy of immune checkpoint inhibitors (36). In prostate cancer, high CXCR2 expression accelerates tumor recurrence, and the CXCR2 inhibitor AZD5069 effectively restrains tumor progression (37).

Notably, accumulating evidence indicates that CXCL2 in the pleural microenvironment originates from multiple cellular compartments rather than a single population. As a canonical ELR^+^ CXC chemokine, CXCL2 is broadly secreted by both hematopoietic and stromal cells under inflammatory and tumor conditions. In the MPE microenvironment, lung adenocarcinoma cells, activated pleural mesothelial cells and cancer-associated fibroblasts have all been identified as important non-myeloid sources of CXCL2, which synergistically build up chemokine gradients to drive myeloid cell recruitment and effusion formation (9, 38). Among myeloid populations, infiltrating neutrophils and polymorphonuclear myeloid-derived suppressor cells represent another major source of CXCL2, forming a paracrine positive feedback loop that further sustains CXCR2 signaling activation and immunosuppressive microenvironment remodeling (39, 40). Therefore, the altered CXCL2 levels observed in our *in vivo* MPE models reflect the combined output of multiple CXCL2-producing cell types, and the independent contribution of non-macrophage sources warrants further quantitative investigation in future studies.

Another critical consideration is the non-specific effects of systemic CXCR2 inhibition across heterogeneous myeloid cell subsets. CXCR2 is not selectively expressed on CD169^high^ macrophages, instead, it is widely distributed on neutrophils, classical monocytes, myeloid-derived suppressor cells and subsets of dendritic cells, all of which are key participants in MPE pathogenesis (41, 42). Pharmacological blockade of CXCR2 therefore exerts broad-spectrum modulatory effects beyond macrophage regulation. For instance, CXCR2 inhibition profoundly impairs neutrophil egress from bone marrow and their intratumoral functional polarization toward an immunosuppressive phenotype, reduces circulating monocyte recruitment and differentiation into tumor-associated macrophages, and attenuates the suppressive activity of myeloid-derived suppressor cells on T cell function (42, 43). Consistent with this, our flow-cytometric analysis showed that SB225002 reduced the absolute number of neutrophils in addition to CD169^high^ macrophages. Therefore, the therapeutic effects of CXCR2 inhibition cannot be attributed exclusively to CD169^high^ macrophages. Instead, our findings support a broader role of CXCR2-dependent myeloid-cell recruitment, within which CD169^high^ macrophages represent an important cellular population associated with MPE progression. This inherent limitation of global pharmacological inhibition highlights the need for subsequent studies using cell-type-specific CXCR2 knockout mouse models to precisely delineate the independent contribution of CD169^high^ macrophages and disentangle the off-target effects on other myeloid lineages.

## Data Availability

The original contributions presented in the study are included in the article/Supplementary Material, further inquiries can be directed to the corresponding author/s.
