# Correction: CXCL2/CXCR2 pathway regulates CD169^high^ large macrophages to promote progression of malignant pleural effusion from lung adenocarcinoma

**DOI:** 10.3389/fmed.2026.2003813

**Published:** 2026-10-05

**Authors:** Sitong Chen, Chunyan Li, Yuezhang Sun, Yanyan Huang, Guifu Gong, Yuxuan Bao, Liyun Xu

**Affiliations:** 1 Department of Clinical Laboratory, Zhoushan Hospital, Wenzhou Medical University, Zhoushan, Zhejiang, China; 2 Cellular and Molecular Biology Laboratory, Zhoushan Hospital, Wenzhou Medical University, Zhoushan, Zhejiang, China; 3 Department of Cardiothoracic Surgery, Zhoushan Hospital, Zhejiang University School of Medicine, Zhoushan, Zhejiang, China; 4 First Affiliated Hospital of Wenzhou Medical University, Wenzhou, China; 5 Department of Pulmonary and Critical Care Medicine, Wenzhou Medical University, Zhoushan, Zhejiang, China

**Keywords:** CD169^+^ macrophages, CXCL2, CXCR2, lung adenocarcinoma, malignant pleural effusion (MPE)

**Incorrect funding**

Funding number incorrect

An incorrect number was provided for [Zhejiang Provincial Medical and Health Science and Technology Plan Project]. The correct number is [2025HY1404].

The original version of this article has been updated.

